# A Novel Platform for Root Protection Applies New Root-Coating Technologies to Mitigate Soil-Borne Tomato Brown Rugose Fruit Virus Disease

**DOI:** 10.3390/v15030728

**Published:** 2023-03-11

**Authors:** Eyal Klein, Elisheva Smith, Chen Klap, Elena Bakelman, Arie Ophir, Aviad Sela, Elena Poverenov, Dmitry Rein, Yachin Cohen, Dan Eliahu, Shai Shahal, Guy Mechrez, Karthik Ananth Mani, Pulikanti Guruprasad Reddy, Abraham J. Domb, Nadav Pass, Aviv Dombrovsky

**Affiliations:** 1 Hishtil Nurseries, Nehalim 4995000, Israel; 2 Department of Plant Pathology and Weed Research, Agricultural Research Organization, The Volcani Institute, Rishon LeZion 7505101, Israel; 3 The Robert H. Smith Faculty of Agriculture, Food and Environment, The Hebrew University of Jerusalem, Rehovot 761001, Israel; 4 Agro-Nanotechnology and Advanced Materials Center, Department of Food Science, Agricultural Research Organization, The Volcani Institute, Rishon LeZion 7505101, Israel; 5 Department of Chemical Engineering, Technion—Israel Institute of Technology, Haifa 3200003, Israel; 6 PolyGreen Ltd., North Industrial Zone, Nahariya 2231103, Israel; 7 School of Pharmacy-Faculty of Medicine, The Hebrew University of Jerusalem, Jerusalem 9112002, Israel

**Keywords:** tobamovirus, soil-mediated virus transmission, soil disinfection

## Abstract

Tomato brown rugose fruit virus (ToBRFV) is a soil-borne virus showing a low percentage of ca. 3% soil-mediated infection when the soil contains root debris from a previous 30–50 day growth cycle of ToBRFV-infected tomato plants. We designed stringent conditions of soil-mediated ToBRFV infection by increasing the length of the pre-growth cycle to 90–120 days, adding a ToBRFV inoculum as well as truncating seedling roots, which increased seedling susceptibility to ToBRFV infection. These rigorous conditions were employed to challenge the efficiency of four innovative root-coating technologies in mitigating soil-mediated ToBRFV infection while avoiding any phytotoxic effect. We tested four different formulations, which were prepared with or without the addition of various virus disinfectants. We found that under conditions of 100% soil-mediated ToBRFV infection of uncoated positive control plants, root-coating with formulations based on methylcellulose (MC), polyvinyl alcohol (PVA), silica Pickering emulsion and super-absorbent polymer (SAP) that were prepared with the disinfectant chlorinated-trisodium phosphate (Cl-TSP) showed low percentages of soil-mediated ToBRFV infection of 0%, 4.3%, 5.5% and 0%, respectively. These formulations had no adverse effect on plant growth parameters when compared to negative control plants grown under non ToBRFV inoculation conditions.

## 1. Introduction

Tobamoviruses are pests that cause highly significant damage to a range of cultivated crops worldwide. Tobamoviruses are seed-borne and highly stable viruses mechanically transmitted via adhesion to surfaces and agro-technical handlings of plants [1,2,3]. Tobamovirus transmission by insect vectors via adhesion of the virus to honeybees and bumblebees has also been documented [4,5,6]. These viruses are also soil-borne and water-borne. Soil is contaminated with tobamoviruses primarily due to virus-infected plant debris buried in soil during consecutive crop growing cycles. Long-term preservation in soil [7,8] and water-mediated virus transmission [9] contribute to virus dispersal to distances far from the primary infected area. The soil-borne tobamoviruses that infect root tips are transmitted to the upper parts of the affected plants [10,11,12,13]. Apart from agro-technique-associated root injury that predisposes roots to viral infections, roots are susceptible to infection through natural wounds in root tips that occur during growth, by changes in turgor or due to the buffering wind [14].

Tobamovirus abundance in soil depends primarily on the soil content of clay, organic matter and water as well as soil temperatures, ionic strength and pH [15]. Tobamoviruses have a net negative charge above the isoelectric point (at pH ~ 3.9) that is confined to one end of the rod-like structure of the virions [16,17,18,19]. Electrostatic attractions occur between tobamoviruses and positively charged sites on clay minerals, which have a high surface area and an anion exchange capacity [20,21,22]. Hydrophobic interactions also occur between tobamoviruses and soil hydrophobic sites. Organic matter reduces tobamovirus adhesion to clay [23]; however, tobamovirus adhesion to organic matter allows the preservation of the virus in water [12]. High ionic strength reduces virus–soil interactions [16] and the presence of divalent cations in the case of tobamovirus adhesion to clay could mask the negative charges on the virions and reduce adhesion as well [24]. High temperatures are correlated with lower viral abundance in soil and soil pH below ~5.0 is generally considered appropriate for good viral adsorption [15,23,25]. Tobamoviruses occur in various water sources and could be transmitted via nutrients in hydroponic systems. The viruses have been detected in drainage water, rivers, forest ditches and brooks and could spread via the irrigation system from infected roots [9,26,27,28].

The tobamovirus tomato brown rugose fruit virus (ToBRFV) has recently been widely dispersed around the world, infecting tomato crops harboring the durable *Tm-2^2^* resistance allele [29,30]. ToBRFV is a single-stranded positive-sense RNA virus (+ssRNA) that encodes four known proteins: two proteins of the replicase complex of 126 kDa and 183 kDa, a movement protein of ~30 kDa and a coat protein (CP) of ~17 kDa [31]. In addition, two putative proteins of 54 kDa and 4–5 kDa have been identified [32,33]. ToBRFV dispersal via infected seeds and fruit mesocarp as well as soil-mediated transmission of the virus to tomato plants have been recently documented [34,35]. We showed that the low number of infectious foci of soil-mediated tobamovirus infection (ca. 1%) could be increased by the addition of a ToBRFV inoculum to the soil and seedling root truncation, providing a small-scale platform for studying soil-mediated ToBRFV infection [35]. However, the preservation of naturally occurring ToBRFV in the soil of ToBRFV-infected crops, infested via the infected roots and the mediation of irrigation water, has not been studied yet. In addition, the management of soil-mediated ToBRFV infection has not been challenged.

Previous work that has shown a profound reduction in soil-mediated tobamovirus infection described the addition of an intermediate clean medium, such as perlite or compost, between deliberately truncated roots and tobamovirus-contaminated soil while planting [36,37,38]. We tested ToBRFV preservation in soil under natural conditions, simulating consecutive growth cycles, and designed a novel platform to counteract soil-mediated ToBRFV transmission to tomato plants via root infection. In our platform, root-coating technologies were designed and assayed for efficiency in hindering soil-mediated ToBRFV plant infection. The efficiencies of the new technologies were tested under rigorous conditions of root truncation and a deliberate soil infestation with ToBRFV. Selected technologies that demonstrated high protection efficiency with a minimal phytotoxic effect are described herein.

## 2. Materials and Methods

### 2.1. Experimental Formats for the Optimization of Soil-Mediated ToBRFV Infection

Tomato plants mechanically inoculated with ToBRFV sap were grown for 30–50 days, in 0.3–1 L pot, or for 90–120 days in 2–10 L pots using a commercial soil medium Green 90 (EvenAri, Beit Elazari, Israel). Following the required time, the plants were removed and the pots with root debris were re-used for the experimental seedlings cvs. Ikram and Antonela for planting. In order to amplify the soil-mediated ToBRFV infection, we implemented two strategies. We added a ToBRFV inoculum from symptomatic tomato plants constantly tested for ToBRFV by enzyme-linked immunosorbent assay (ELISA) using ToBRFV-specific antiserum to soil pits. In addition, RT-PCR followed by amplicon Sanger sequencing was routinely conducted. An inoculum of ToBRFV was prepared by grinding 2 kg of infected tomato leaves in 10 L 0.1 M sodium phosphate buffer with pH = 7.0. The inoculum was diluted 1:5 with tap water at the experimental greenhouse and planting pits of ~50 mL each were filled with ToBRFV inoculum source. In addition, we truncated seedling root tips to increase the susceptibility to ToBRFV infection [35]. 

### 2.2. Root Coating Technologies

For carboxymethylcellulose (CMC)-based coating, CMC was dissolved in deionized water while stirring on a hot plate stirrer at 80 °C for two hours to obtain a 1% (*w*/*v*) solution. For methylcellulose (MC)-based coating, MC powder was dissolved in deionized water while stirring on a hot plate stirrer at 50–60 °C for two hours, and then left to stir at room temperature for 10 h to obtain a 1.5% (*w*/*v*) solution. Sodium dichloroisocyanurate (NaDCC) powder or chlorinated trisodium phosphate (Cl-TSP, 97% TSP, 3% Cl) was dissolved while stirring for 10 min in the polymer solution to obtain concentrations of 2% and 3% (*w*/*v*) solutions, respectively.

For polyvinyl alcohol (PVA)-based coating, PVA of 36 kDa (Sigma Aldrich Co.) was dissolved while stirring on a hot plate stirrer in 85–90 °C tap water for 10 min to obtain a 5% (*w*/*w*) aqueous solution. Formulations with NaDCC or Cl-TSP were prepared by dissolving the viricidal substances in an obtained solution of PVA under continuous stirring for 5 min at room temperature to avoid the premature decomposition of the viricides. 

For silica or PVA Pickering emulsion-based coating, Pickering emulsions contained 1 wt% silica (R812 of Evonic, Germany) or 1% PVA at an oil/water phase ratio of 50:50. The oil phase was canola oil. Emulsification was performed using a high shear mixer (IKA ultra turrax). Then, 0.5% sodium polyacrylic acid (SPA) and different concentrations (2, 3%) of Cl-TSP or different concentrations of NaDCC were dissolved in the water before emulsification.

For Polygreen’s Super Absorbent Polymer, named Plantov SAP (SAP)-based coating, SAP was diluted in water at concentrations of 0.5%, 0.75% and 1%. When adding Cu-Thymol (copper thymol, 15%), SAP and the salt were mixed in water until homogenous hydrogel was obtained. Similarly, 3% Cl-TSP was prepared with the SAP. Formulations involving CuCl2, DOPA-Phe-NH2 with CuCl2 in a complex (3:1), copper phosphate salt, 15% potassium phosphate salt (MPP), 20% ammonium phosphate salt (DAP) and mustard oil (MO) were mixed according to percentile weight in Plantov SAP as a custom fit process during manufacturing. The formulas were dried and ground to form homogeneous dry granules. The mixture was stirred in water until homogeneous hydrogel was obtained.

For Cu-thymol synthesis, Cu-thymol was synthesized as per a previous report [39]. In addition, the copper content in the Cu-thymol was also quantified based on the Zincon method [40] from the UV-visible analysis as 17.4%. Briefly, to a 1 gm/10 mL methanol solution of Thymol (1eq, 6.656 mmol), a NaOH solution of 0.266 gm/2 mL water (1eq, 6.656 mmol) was added. The reaction mixture was stirred at RT for 12 h. After completion of the reaction, the solvent was evaporated and the residual Na-thymol was crystalized in n-heptane to obtain a 1.2 g semi-solid. Cu-thymol was obtained from the reaction of Cu(II)SO4.5H2O and Na-Thymol at a 1:2 mole ratio in ethanol water to form a dark green precipitate. 

### 2.3. Application of the Tested Root Coating Technologies

For soil infestation, the ToBRFV inoculum was poured into soil pits, which were irrigated by sprinklers for 10 min and then left for 1–2 h prior to the experimental planting. During 2020–2021, seedlings of tomato plants cvs. Ikram and Zohara were used for optimization of root coating formulation experiments. Seedlings of tomato plants cv. Tori were used (September 2021) for a comparative root coating efficiency study on mitigating ToBRFV soil-mediated infection and affecting plant growth. Seedlings were truncated and root-coated (controls were uncoated) by dipping the truncated roots in the coating solutions before planting. 

### 2.4. Serological Assays

Western blot analyses and ELISA tests were conducted as previously described [34]. For western blot of the virion preparations, USB buffer containing 75 mM Tris-HCL (pH = 6.8), 8 M urea, 4.5% (g/v) SDS and 7.5% (*v/v*) ß-mercaptoethanol were added in addition to Laemmli sample buffer [41]. The employed antibodies were raised against ToBRFV virions purified from virus-infected *Tm-2*^2^-resistant tomato plants [29] and used in dilution ratios of 1:4000 in PBS. For ELISA tests, similar leaf sizes were sampled. ToBRFV ELISA-positive infections had optical density (O.D.) values at least 3.0 times greater than the negative controls. 

### 2.5. Soil Virion Purification

Soil was collected from the pots, weighed and ca. two equivalent volumes of 0.1 M sodium phosphate buffer pH = 7.0 were added. The suspensions were stirred overnight at 4 °C and centrifuged at ~1500× *g* for 20 min followed by supernatant filtration through a gauze. Centrifugations and filtrations were repeated three times before ultracentrifugation of the supernatants was conducted at 186,000× *g* for 3 h at 6 °C. Pellets were re-suspended in 0.01 M sodium phosphate buffer pH = 7.0.

### 2.6. Statistical Analyses

Statistical analyses for the challenging experimental format were undertaken using JMP Pro 16 (SAS Institute Inc.). Comparisons of soil-mediated ToBRFV infections were carried out on the arcsine-transformed square root proportions of positively infected plants and subjected to an analysis of standard least squares by the restricted maximum likelihood (REML) method followed by Tukey’s HSD test for multiple comparisons among means. In the statistical analysis, the variable of tested plants in each repeat was nested in the variable of repeats and both were assigned as random effects, as these could not be regarded as independent variables. Treatments were used separately as variables to examine the interactions between the different treatments. Comparisons of root coating treatments with negative control or positive ToBRFV-infected control were conducted by analyses of standard least squares by the REML method followed by post hoc Dunnett’s test.

## 3. Results

### 3.1. Developing a Biological Assay for Challenging Technologies of Root Protection

**Figure 1:**
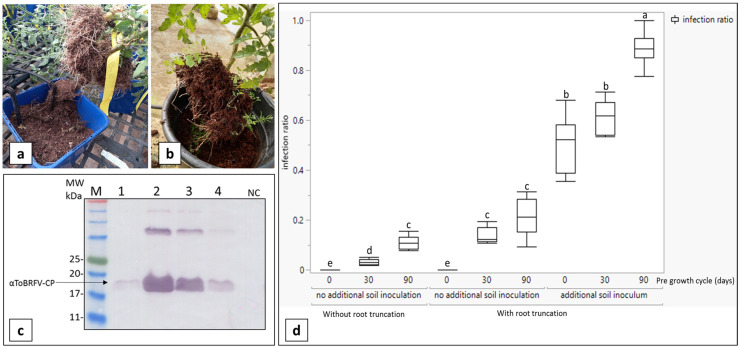
Modes of soil-mediated ToBRFV infectivity potential. (**a**,**b**) Pots with root debris from ToBRFV-infected plants of pre-growth cycles of 90–120 days. (**c**) Western blot analysis of ToBRFV virions isolated from four individual pots with infected root debris. M, molecular size marker; NC, negative control; CP, coat protein. (**d**) Efficiency comparison of ToBRFV pre-growth cycle time length, a ToBRFV soil inoculum and root truncation. Boxes are interquartile ranges with horizontal lines indicating the 25th, median and 75th percentiles. Statistical analysis was performed on the arcsine square root-transformed values in terms of standard least squares by restricted maximum likelihood (REML) followed by Tukey HSD. Different connecting letters designate significant differences between treatments (α < 0.05). Zero infection ratios were observed in negative control plants grown in non-inoculated soil without pre-growth cycle root debris.

We previously showed that ToBRFV soil inoculation and root truncation increased the infectivity percentage of soil-mediated ToBRFV infection in positive control plants and, accordingly, small-scale experiments could be conducted [35]. In order to further increase the infectivity percentage of soil-mediated ToBRFV infection of positive control plants, we now extended our study and added a factor of root debris from the pre-growth cycle of ToBRFV-infected tomato plants. Tomato plants infected by mechanical ToBRFV sap inoculation were grown for 30–50 days or 90–120 days. The plants were removed, leaving root debris in the planting pots (Figure 1a,b). ToBRFV virions in the soil were isolated and analyzed by western blot (Figure 1c). Plants grown in pots containing a 30 day root debris pre-growth cycle showed a ToBRFV infectivity percentage of 3.27% ± 1.1 standard deviation (S.D.), n = 30–84 (a total of 538 plants). Plants grown in pots containing 90 day root debris pre-growth cycles showed ToBRFV infectivity percentages of 88.5% ± 6.5 S.D., n = 15–58 (a total of 410 plants). Analyses of the various modes of soil-mediated infectivity potential revealed significant differences between the duration of previous cycle of growth (DF = 2, F = 42.15, *p* < 0.0001). The soil inoculation effect was significant (DF = 1, F = 265.33, *p* < 0.0001), as well as root truncation (DF = 1, F = 13.63, *p* = 0.0007). The interaction between the duration of the previous cycle, soil inoculation and root truncation was found to be significant (DF = 2, F = 7.076, *p* = 0.0118 and DF = 2, F = 4.17, *p* = 0.0229, respectively). Combining the entire set of available treatments resulted in significant differences between combinations (DF = 8, F = 26.85, *p* < 0.0001; Figure 1d). As expected, the best performing treatment for ToBRFV soil-mediated infection included ninety-day previous cycle duration accompanied by both soil inoculation and root truncation (*p* = 0.0001). However, if both soil inoculation and root truncation were conducted, no significant differences between zero days and thirty days were observed (*p* = 0.86). Apparently, root truncation had a significant effect on ToBRFV soil-mediated infection but has the lowest effect when compared with the time length of the pre-growth cycle and soil inoculation (Figure 1d).

### 3.2. Root Coating Formulations for Root Protection against Soil-Mediated ToBRFV Infection

**Figure 2:**
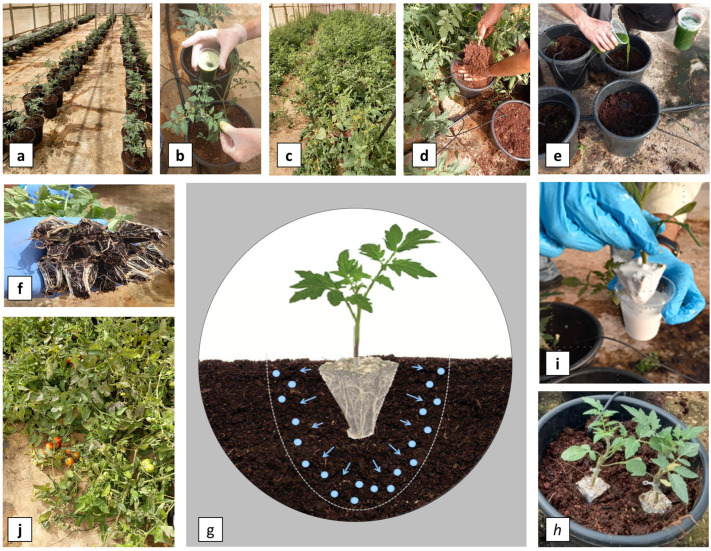
Illustration of the root coating experiments. (**a**–**c**) Pre-growth cycle of ToBRFV-infected tomato plants for 120 days. (**d**) Plant removal while root debris remained in the soil medium. (**e**) Adding a ToBRFV inoculum into the planting pits. (**f**) Root truncation. (**g**) An illustration of the root coating protection layer loaded with disinfectants. (**h**) Dipping the truncated roots in root coating formulations. (**i**) Planting the root-coated seedlings. (**j**) Plant growth up to the first fruit cluster harvest.

Four selected technologies were tested for their potential to mitigate ToBRFV soil-mediated infection under conditions of our developed format (Figure 2). Our experimental format included root debris from ~30 day or ~90 day pre-growth cycles of ToBRFV-infected plants (Figure 2a–d); additionally, a ToBRFV inoculum was added to the reused soil (Figure 2e), root truncation was performed prior to dipping the roots in the tested root coating formulations (Figure 2f–h) and the plants were planted and grown until the fruit harvesting stage (Figure 2i,j). At first, we tested the various formulations for root coating to mitigate soil-mediated ToBRFV infection occurring in soil containing 30–50 day pre-growth cycle debris (Table 1). The range of positive control infection ratios was 46.4–100%. 

The low number of experiments for each formulation and the high range of plant numbers in the experiments only permit the following deductions regarding the relative contributions of root-coating formulations within each group of experiments:

**MC-based emulsion:** MC alone demonstrated low efficiency in reducing ToBRFV infection; however, when combined with Cl-TSP 14.2% infection was observed, the effectivity of the protection increased by 85.8 % (a factor of five);

**PVA-based emulsion:** The combination of PVA with Cl-TSP or NaDCC showed 7% and 4.7% ToBRFV infection, respectively. The two formulations were effective in protection against ToBRFV infection, showing effectivity of 93% and 95.3%, respectively;

**Silica and PVA Pickering emulsions:** In one experiment, silica Pickering emulsion alone was effective in root protection. The formulations of either silica or PVA Pickering emulsions with Cl-TSP and NaDCC showed high protection efficiencies of 95–97% and 86–89%, respectively;

**SAP-based formulations:** SAP alone (0.75%) showed a high protective efficiency of 89%, while 0.5% SAP was not as effective but the addition of Cu-Thymol increased its protection efficiency by a factor of 1.7. Formulations of 0.75% SAP with mustard oil showed a high efficiency of 82% of plant protection against ToBRFV infection. 

### 3.3. Comparative Effects of the Various Root Coating Technologies on Tomato Plant Growth and Susceptibility to Soil-Mediated ToBRFV Infection

The growth parameters of survival, foliar biomass per plant and fruit yield per plant were assessed in response to root coating treatments under two conditions of either a regular growth or growth in ToBRFV-inoculated soil containing root debris of 120 days from pre-grown ToBRFV-infected tomato plants. The efficiency of mitigating ToBRFV soil-mediated infection of the root-coated plants was measured using an ELISA test.

**Figure 3:**
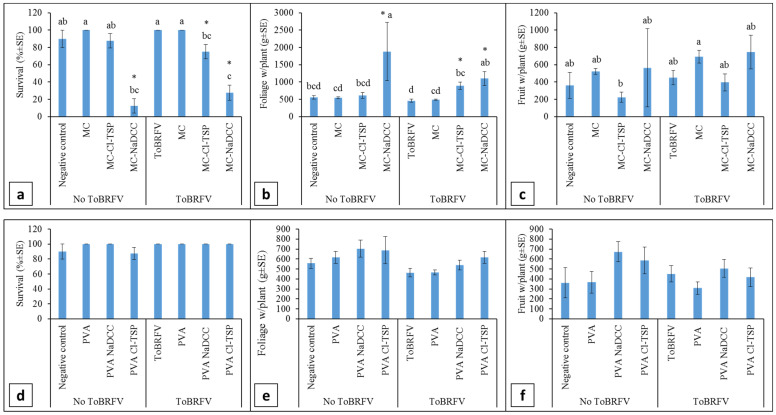
The effects of MC (**a**–**c**)- and PVA (**d**–**f**)-based coatings on plant growth grown in non-inoculated or ToBRFV-inoculated soil. (**a**) Plant survival. MC + NaDCC reduced survival: c vs. a. Plants in ToBRFV-inoculated soil coated with MC + Cl-TSP showed reduced survival: bc vs. a, which was not significant when compared to the negative control. (**b**) Foliar biomass per plant. MC + Cl-TSP and MC + NaDCC showed increased levels: bc, ab vs. d. (**c**) Fruit weight per plant. MC alone in plants grown in ToBRFV-inoculated plants showed increased levels when compared with MC + Cl-TSP in plants grown in non-inoculated plants: a vs. b. (**d**) Plant survival. (**e**) Foliar biomass per plant. (**f**) Fruit weight per plant. Statistical analysis was conducted using standard least squares by REML. Different connecting letters designate significant difference between treatments tested by post hoc Tukey HSD (α < 0.05). Asterisks denote significant differences following comparisons with controls using post hoc Dunnett (α < 0.05).

**MC-based coating:** The effects of MC-based root coatings on tomato plants’ growth parameters are illustrated in Figure 3a–c. Clearly, ToBRFV soil inoculation did not reduce survival when comparing negative control plants and the ToBRFV-infected positive control plants (Figure 3a, n = 9–20). In addition, MC and MC + Cl-TSP did not affect the survival of plants grown under regular conditions of non-inoculated soil. However, MC + NaDCC reduced the survival of plants grown under both non-inoculated and ToBRFV-inoculated conditions. When compared with the negative control plants, significance values were *p* = 0.0242 and *p* = 0.0015 for MC + NaDCC root-coated plants grown in non-inoculated and ToBRFV-inoculated soil, respectively, n = 9–20. The reduction in survival by MC + Cl-TSP of plants grown in ToBRFV-inoculated soil, although lower, was also significant when compared with positive control uncoated ToBRFV-infected plants *p* = 0.0065, n = 15–20. However, MC + Cl-TSP’s effect on survival was not significantly lower than that of the control plants grown in non-inoculated soil, n = 9–15. Foliar biomass per plant was not changed when uncoated plants were grown in ToBRFV-inoculated soil compared to negative control plants grown in non-inoculated soil (Figure 3b). However, root coating by MC + NaDCC showed a higher foliar biomass per plant in plants grown in both non-inoculated and in ToBRFV-inoculated soil; when compared to negative control plants, the significance values were *p* = 0.001 and *p* = 0.02, respectively, n = 9–20. MC + Cl-TSP in root-coated plants grown in ToBRFV-inoculated soil showed high foliar biomass per plant when compared with the positive controls with *p* = 0.0095, n = 13–20, but not when compared with negative control plants. Fruit weight per plant showed no differences between negative control plants grown in non-inoculated soil and the positive uncoated control plants grown in ToBRFV-inoculated soil (Figure 3c). High fruit weight per plant was observed in MC root-coated plants grown in ToBRFV-inoculated soil compared with MC + Cl-TSP root-coated plants grown in non-inoculated soil but there was no significant difference when comparing the root-coated plants with the negative control plants or with the positive ToBRFV-infected control plants. Under the same experimental conditions of ToBRFV-inoculated soil containing root debris of ca. 90 day pre-grown ToBRFV-infected plants, plants showed 100% infection when tested using ELISA (n = 21). Root coating with MC alone (1.5%) showed 19.1% infection, whereas 1.5%MC + 3%Cl-TSP and 1.5%MC + 2%NaDCC each showed 0% infection (n = 5–21) (Table 2).

**PVA-based coating:** Plant survival, foliar biomass per plant and fruit weight per plant were not affected by ToBRFV inoculation of the soil containing root debris of ca. 90 days from pre-grown plants, as shown in Figure 3d–f. PVA-based root coating, including PVA + NaDCC and PVA + Cl-TSP, did not affect any of the growth parameters that were tested. ELISA test of the root-coated plants showed that, in a background of 100% infection of plants grown in the ToBRFV-inoculated soil (n = 21), PVA (5%) alone, 5%PVA + NaDCC (containing about 400 ppm of active chlorine, which corresponds approximately to 2% of very fresh NaDCC) and 5%PVA+ 3%Cl-TSP were measured as 13.3%, 10.5% and 4.3% in ToBRFV-infected plants (n = 15–23) (Table 2).

**Figure 4:**
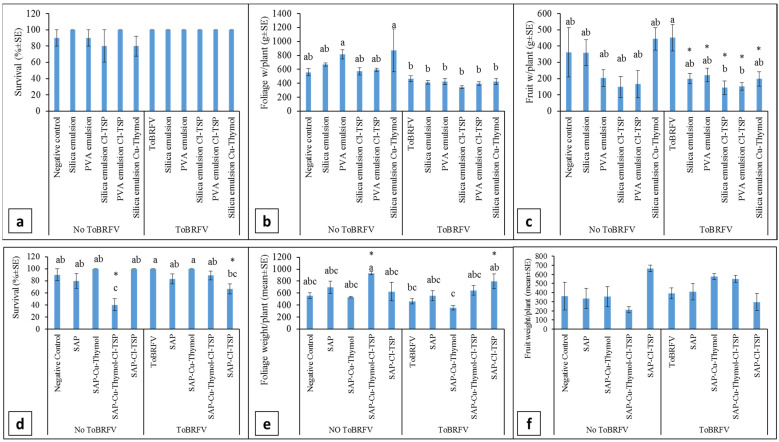
The effects of silica or PVA Pickering emulsion-based coating (**a**–**c**) and SAP-based coating (**d**–**f**) on plants grown in non-inoculated or ToBRFV-inoculated soil. (**a**) Plant survival. (**b**) Foliar biomass per plant. PVA and silica + Cu-Thymol Pickering emulsions showed high levels in plants grown in non-inoculated soil compared with plants grown in ToBRFV-inoculated soil: a vs. b. (**c**) Fruit weight per plant. Silica + Cl-TSP and PVA + Cl-TSP Pickering emulsion root coating showed reduced levels in plants grown in ToBRFV-inoculated soil compared with uncoated pants: b vs. a, but there was no reduction when compared to negative control plants grown in non-inoculated soil: b vs. ab. (**d**) Plant survival. SAP + Cu-Thymol + Cl-TSP reduced survival in plants grown in non-inoculated soil: c vs. ab, but not in plants grown in ToBRFV-inoculated soil. SAP + Cl-TSP showed lower survival in plants grown in ToBRFV-inoculated soil: bc vs. a, but the survival percentage was similar to control plants grown in non-inoculated soil. (**e**) Foliar biomass per plant. SAP + Cu-Thymol + Cl-TSP showed high levels in plants grown in non-inoculated soil compared with SAP + Cl-TSP: a vs. bc, but there was no difference when compared to control plants. SAP + Cl-TSP showed higher levels in plants grown in ToBRFV-inoculated soil when compared with SAP + Cu-Thymol: ab* vs. c. SAP + Cl-TSP showed higher levels in plants grown in ToBRFV inoculated soil compared with ToBRFV-infected uncoated plants: ab*, but there was no difference when compared with negative control plants. (**f**) Fruit weight per plant. No significant differences were observed. Statistical analysis was conducted using standard least squares by REML. Different connecting letters represent significant differences between treatments by host hoc Tukey HSD (α < 0.05). Asterisks denote significant differences in comparisons with controls using post hoc Dunnett (α < 0.05).

Silica or PVA Pickering emulsion-based coating: The effects of silica and PVA-based Pickering emulsions used for root coating on plant growth parameters is illustrated in Figure 4a–c. ToBRFV soil inoculation did not affect plant survival, foliar biomass per plant and fruit weight per plant when comparing uncoated ToBRFV-infected plants with uncoated plants grown in non-inoculated soil. Root coatings with silica or PVA Pickering emulsion-based formulations did not affect plant survival. Root coating with PVA and silica + Cu-Thymol Pickering emulsions showed higher foliar biomass per plant in plants grown in non-inoculated soil compared with plants grown in ToBRFV-inoculated soil. These two root coating formulations, PVA and silica + Cu-Thymol Pickering emulsions, showed high foliar biomass per plant when compared with ToBRFV-infected control plants, *p* = 0.0093 and *p* = 0.0016, respectively, n = 9–20, but there was no difference when compared to negative control plants grown in non-inoculated soil (Figure 4b). Silica + Cl-TSP and PVA + Cl-TSP Pickering emulsion-based root coatings showed reduced fruit weight per plant when comparing plants grown in ToBRFV-inoculated soil with the ToBRFV-infected positive control (Figure 4c). A comparison with ToBRFV-infected positive control plants showed that fruit weight per plant of silica, PVA, silica + Cl-TSP, PVA + Cl-TSP and silica + Cu-Thymol root coated plants grown in ToBRFV-inoculated soil as well as silica + Cl-TSP and PVA + Cl-TSP root-coated plants of non-inoculated soil were each lower than the positive control plants with *p* = 0.015, *p* = 0.033, *p* = 0.001, *p* = 0.002, *p* = 0.014, *p* = 0.036 and *p* = 0.029, respectively, n = 18–20. However, there was no significant reduction in fruit weight per plant when comparing all root-coated plants with the control plants grown in non-inoculated soil, n = 9–18. ELISA was used to test the root coating effect on ToBRFV infection compared to 100% infection of the uncoated controls (n = 21) and showed that silica, PVA, silica + 3%Cl-TSP, PVA + 2%Cl-TSP and silica + 15%Cu-Thymol Pickering emulsions showed 5.9%, 22.2%, 5.6%, 26.3% and 10.5% of infections respectively, n = 17–19 (Table 2).

SAP-based coating: Plants grown in ToBRFV-inoculated soil showed no difference in survival, foliar biomass per plant and fruit weight per plant when comparing the uncoated positive control plants to control plants grown in non-inoculated soil (Figure 4d–f). SAP coatings did not affect these three parameters as well. However, SAP + Cu-Thymol + Cl-TSP showed a significant reduction in survival of plants grown in non-inoculated soil. The lower levels caused by SAP + Cu-Thymol + Cl-TSP were significant when compared with either negative control plants or ToBRFV-infected uncoated positive control plants with *p* = 0.0002 and *p* = 0.0001, respectively, n = 5–9. This reduction in survival did not occur in SAP + Cu-Thymol + Cl-TSP root-coated plants grown in ToBRFV-inoculated soil. SAP + Cl-TSP showed reductions in the survival of plants grown in ToBRFV-inoculated soil when compared with positive control ToBRFV-infected plants with *p* = 0.0008 n = 12–20, but there was no significant change when compared to negative control plants (Figure 4d). Foliar biomass per plant showed an increase in plants root-coated with SAP + Cu-Thymol + Cl-TSP that were grown in non-inoculated soil and SAP + Cl-TSP root coated plants grown in ToBRFV-inoculated soil; the latter was higher than in plants root coated with SAP + Cu-Thymol (Figure 4e, ab* vs. c). A significant increase in foliar biomass per plant was observed when comparing SAP + Cu-Thymol + Cl-TSP root-coated plants grown in non-inoculated soil and SAP + Cl-TSP root-coated plants grown in ToBRFV-inoculated soil with the positive control ToBRFV-infected uncoated plants showing *p* = 0.01 and *p* = 0.018, respectively, n = 5–16. However, there was no significant difference when comparing all root-coated plants with the negative control plants grown in non-inoculated soil. No significant differences were observed between all root-coated and uncoated control plants in fruit weight per plant (Figure 4f). ELISA test of the infected plants showed that in a background of 100% ToBRFV infection (n = 21), SAP alone (0.75%) showed 0% infection and 0.75%SAP + 15%Cu-Thymol, 0.75%SAP + 15%Cu-Thymol + 3–5%Cl-TSP and 0.75%SAP + 3–5%Cl-TSP showed 17.6%, 0% and 0% infection, respectively, n = 14–23 (Table 2).

## 4. Discussion

We employed rigorous and challenging experimental conditions to test a new platform of root protection against soil-mediated ToBRFV infection of tomato plants harboring the *Tm-2*^2^ resistance allele. In face of the low infectious potential of soil-mediated tobamovirus infection, we previously showed that virus inoculation of the soil in addition to root truncation prior to planting increased the susceptibility of tomato plants to tobamovirus infection [35] and reduced the plant number necessary to draw conclusions regarding soil disinfection treatments. Here we improved the conditions for ToBRFV infection by using soil containing root debris left from ToBRFV-infected tomato plants that were grown for 90–120 days prior to the new seedling planting. Under these stringent conditions, the virus infected all plants that were planted in the ToBRFV-inoculated soil containing the root debris. Our analysis of the new conditions revealed that a longer pre-growth cycle combined with ToBRFV soil inoculation were the critical parameters to ensure high soil-mediated ToBRFV infection efficiency. Root truncation had a significant effect on accelerating infections but it had the lowest effect compared with the time of the pre-growth cycle and ToBRFV soil inoculations (Figure 1). 

The new platform of root protection involves root coating by an insulating chemical emulsion that was tested alone or in the presence of disinfectants. A similar concept was originally performed by employing an intermediate medium technology [36,37,38]; however, the volumes used for root coating are low and could be adapted to large-scale applications.

The effect of root coating on the mitigation of soil-mediated ToBRFV infection was tested in plants grown in soil with infected root debris of pre-grown ToBRFV-infected plants with the addition of a ToBRFV inoculum. Root truncation was conducted before dipping the roots in the coating formulation. These conditions were highly challenging for the root coating efficiency test in protecting plants against soil-mediated ToBRFV infection. In addition, the presence of organic ingredients in the root debris reduced the efficiency of chlorinated disinfectants [42,43], which were included in the root coating formulations. Several of the disinfectants that were used in the formulations were already known as effective against tobamoviruses such as TSP [2,44,45], Cl-TSP [35], NaDCC [28,35] and Thymol [46]. Copper is also known to have antiviral properties in general [47].

Four groups of root-coating technologies were presented. 

MC-based coating: Testing CMC- and MC-based coatings as formulations for mitigating soil-mediated ToBRFV infection (Table 1) showed that MC was clearly superior. However, the addition of NaDCC caused a prominent reduction in plant survival, being that nature-sourced polysaccharide MC is more prone to oxidation reactions. Thus, the reaction of NaDCC with MC can lead to decomposition of this biopolymer to additional oxidative species that can negatively affect plant survival. However, the effects on foliar biomass per plant and fruit weight per plant in plants grown in ToBRFV-inoculated soil were not reduced compared to the uncoated positive control plants. The results showed that root coatings with MC-NaDCC increased the foliar biomass of the plants when compared with either positive uncoated control or the negative control (Figure 3a,b). These results might biased because the nutrient availability in the pots were planted with two seedlings per pot (Figure 2i) and one of the plants died. In the presence of NaDCC, there was no ToBRFV infection in the five surviving plants tested using ELISA. Unlike NaDCC, MC + Cl-TSP, which showed a reduction in the survival of root-coated plants grown in ToBRFV-inoculated soil compared with positive control plants, the lower levels were not significantly different from control plants grown in non-inoculated soil (Figure 3a). ELISA test of MC + Cl-TSP’s effect on the mitigation of soil-mediated ToBRFV infection showed no infection in thirteen plants compared with 100% infection in the uncoated positive control plants. MC alone increased fruit weight per plant when compared with the MC + Cl-TSP of plants grown in non-inoculated soil but there was no significant difference when compared to the negative control plants (Figure 3c). ELISA test of the efficiency of MC and of MC + Cl-TSP in mitigating soil-mediated ToBRFV infection showed 19.1% and 0% infection, respectively, under conditions of 100% infection of the uncoated positive control plants.

PVA based coating: In face of the mild effects of Cl-TSP on tomato plants’ growth [35] and the similar mitigation effects of ToBRFV infection by PVA + Cl-TSP and PVA + NaDCC (Table 1), PVA + Cl-TSP was the preferable choice for further studies. Using PVA had no ill effect on plant survival even in the presence of NaDCC (Figure 3d). Apparently, root coating by this formulation protected the plants from the phytotoxic effect of NaDCC in addition to mitigation of soil-mediated ToBRFV infection. There were no differences between root-coated plants and uncoated control plants in foliar biomass per plant and in fruit weight per plant (Figure 3e,f). ELISA test for efficiency of mitigation of soil-mediated ToBRFV infection by PVA + Cl-TSP showed a low 4.3% infection percentage compared with 100% infection of the control uncoated plants.

Silica or PVA Pickering emulsion-based coating: Root coatings with either silica or PVA based Pickering emulsions similarly mitigated soil-mediated ToBRFV infections excluding the emulsions in the presence of NaDCC (Table 1). Apparently, silica alone has a positive effect on mitigating soil-mediated ToBRFV infection, acting as a physical barrier. The presence of Cl-TSP might have an additional effect in long-term applications. Both silica and the PVA-based Pickering emulsion root coating did not reduce plant survival (Figure 4a). Root coating with either silica + Cu-Thymol or PVA Pickering emulsions increased foliar biomass per plant in plants grown in non-inoculated soil when compared with the ToBRFV-infected positive control plants (Figure 4b). Root coating with silica and PVA Pickering emulsions with or without Cl-TSP as well as silica + Cu-Thymol showed lower levels of foliar biomass per plant in plants grown in ToBRFV-inoculated soil compared with uncoated ToBRFV-infected positive control plants; however, there was no significant reduction when compared to uncoated control plants grown in non-inoculated soil (Figure 4b). Measuring fruit weight per plant showed that all silica and PVA Pickering emulsion-based root-coated plants had lower levels when compared with the positive uncoated ToBRFV-infected control, but there was no significant reduction when the treated plants were compared with the negative uncoated control plants grown in non-inoculated soil (Figure 4c). Root coating with silica-based emulsions showed high efficiency in mitigating soil-mediated ToBRFV infection, i.e., ~6% infection in the ELISA test compared with 100% infection of the positive non-inoculated control.

SAP-based coating. Root coating with SAP alone showed promising results of 11% ± 13 ToBRFV infection, although the soil had of 30-day pre-growth cycle debris (Table 1). SAP-based coating using SAP + Cu-Thymol + Cl-TSP showed reduced plant survival compared with negative uncoated controls in plants grown in non-inoculated soil but not in plants grown in ToBRFV-inoculated soil (Figure 4d). SAP + Cl-TSP root coating showed lower percent survival in plants grown in ToBRFV-inoculated soil when compared with positive uncoated controls but there was no significant reduction when the results were compared with negative control plants grown in non-inoculated soil (Figure 4d). Measuring foliage weight per plant showed that SAP + Cl-TSP root coatings had high values in plants grown in ToBRFV-inoculated soil when compared with positive uncoated control plants (Figure 4e). Root coatings with SAP-based coating did not affect fruit weight per plant in plants grown in ToBRFV-inoculated soil and in plants grown in non-inoculated soil (Figure 4f). The efficiencies of mitigation of soil-mediated ToBRFV infection measured using ELISA were 0% infection when using SAP, SAP + Cu-Thymol + Cl-TSP and SAP + Cl-TSP compared with 100% infection of the positive uncoated controls.

The presented root-coating formulations are apparently promising, showing high efficacy of plant protection against tobamovirus soil-mediated infection. We did not detect, however, any growth-promoting effect of emulsions based on cellulose, PVA and silica, although cellulose-based biopolymers accelerated shoot elongation, PVA improved plant growth and silica stimulated plant growth [48,49,50]. Any basal growth promoting effects of these emulsions may be dependent on planting soil and/or might be apparent in longer experimental durations. In our experiments, we found that the formulations did not have any adverse effect on plant growth parameters. Mechanization of the root-coating technology by coating trays in the nurseries could serve as a necessary step towards the large-scale upgrading of root protection. Mitigation of soil-mediated tobamovirus infections, under natural conditions of consecutive growth cycles, could reduce the foci of the primary source of infections and hence dramatically affect the secondary spread of tobamovirus. Engaging naturally occurring disinfectants or any commercially available biocids with the various formulations could upgrade the root coating efficiency for a broad range of soil pathogens in large-scale applications. 

## 5. Conclusions

ToBRFV soil-mediated infection is 1–3% and could be enhanced to 80–100% by using reused ToBRFV-contaminated soil, adding ToBRFV inoculum to planting pits and truncating roots, which predisposes seedlings to soil-mediated infections. 

Root coating technology can contribute to the protection of seedlings against soil-mediated ToBRFV infection and could be extended to other tobamoviruses and soil-borne viruses in general.

Formulations based on MC, PVA, silica Pickering emulsion and SAP, prepared in the presence of Cl-TSP for root coating, were highly effective in the mitigation of soil-mediated ToBRFV infection.

## Figures and Tables

**Table 1:** Optimal formulations/emulsions with active chemicals for root protection.

Treatment	Infection Ratio * (%) ± S.D. **	No. of Exp.	No. of Plants/Treatment
MC or CMC based coating			
Negative control (no treatment)	0	2	10–15
1.5%MC	82.6 ± 17.9	2	10–41
1.5%MC + 3%Cl-TSP	14.2 ± 17.3	5	8–41
1.5%MC+ 2%NaDCC	70	1	10
1%CMC	47.8 ± 3.1	2	10–41
1%CMC + 3%Cl-TSP	30.6 ± 27.4	2	10–41
1%CMC + 2%NaDCC	50	1	10
PVA-based coating			
PVA + 2%NaDCC	4.7 ± 6.6	2	11–50
PVA + 3%Cl-TSP	7.0 ± 12.1	4	7–50
Negative control (no treatment)	0	2	15
Silica or PVA Pickering emulsion-based coating			
Silica	0	1	20
Negative control (no treatment)	0	2	10–20
Silica + 3%Cl-TSP	2.8 ± 6.2	5	9–20
Silica+ 2%NaDCC	14.1 ± 1.2	2	20
PVA	13.3	1	20
PVA + 2%Cl-TSP	4.7 ± 4.1	3	18–20
PVA + 2%NaDCC	11.1 ± 15.8	2	20
3% Cl-TSP	5.0 ± 7.1	2	20–21
SAP-based coating			
0.5% SAP	57.1	1	40
0.5% SAP + 5%Cu-Thymol	28.6	1	40
1.0% SAP	57.1	1	40
1.0% SAP + 5%Cu-Thymol	57.1	1	40
Negative control (no treatment)	0	1	30
0.75% SAP	11.0 ± 13.1	4	7–12
0.75% SAP + 0.4%DOPA + 1.2%CuCl2	71	1	12
0.75% SAP + 1.2%CuCl2	71	1	12
0.75%SAP + 15%Copper phosphate	71	1	12
0.75% SAP + 15%MPP	53.9	1	12
0.75% SAP + 20%DAP	53.9	1	12
0.75% SAP + 15%MO	36	1	12
0.75% SAP + 30%MO	18	1	12
Negative control (no treatment)	0	1	12

* Infection ratio equals the infection percentage of treated plants divided by the respective positive control. ** S.D., standard deviation.

**Table 2:** A comparative root coating efficiency effect against ToBRFV soil-mediated infection.

Root Coating Formulation	No. Plants	No. Infected	% Infection
Positive control (uncoated roots)	21	21	100
Negative control	25	0	0
1.5%MC	21	4	19.05
1.5%MC + 3%Cl-TSP	13	0	0.00
1.5%MC + 2%NaDCC	5	0	0.00
5%PVA	15	2	13.33
5%PVA + 2%NaDCC	19	2	10.52
5%PVA+ 3%Cl-TSP	23	1	4.34
Silica Pickering emulsion	17	1	5.88
PVA Pickering emulsion	18	4	22.22
Silica Pickering emulsion + 3%Cl-TSP	18	1	5.56
PVA Pickering emulsion + 2%Cl-TSP	19	5	26.32
Silica Pickering emulsion + 15%Cu-Thymol	19	2	10.53
0.75%SAP	16	0	0
0.75%SAP + 15%Cu-Thymol	17	3	17.61
0.75% SAP + 15%Cu-Thymol + 3%Cl-TSP	14	0	0
0.75% SAP + 3%Cl-TSP	10	0	0

## Data Availability

Not applicable.
